# An intrinsically disordered transcription activation domain increases the DNA binding affinity and reduces the specificity of NFκB p50/RelA

**DOI:** 10.1016/j.jbc.2022.102349

**Published:** 2022-08-05

**Authors:** Hannah E.R. Baughman, Dominic Narang, Wei Chen, Amalia C. Villagrán Suárez, Joan Lee, Maxwell J. Bachochin, Tristan R. Gunther, Peter G. Wolynes, Elizabeth A. Komives

**Affiliations:** 1 Department of Chemistry and Biochemistry, University of California, San Diego, La Jolla, California, USA; 2 Department of Chemistry and Center for Theoretical Biological Physics, Rice University, Houston, Texas, USA

**Keywords:** DNA-protein interaction, cooperativity, hydrogen-deuterium exchange, intrinsically disordered protein, NF-kB transcription factor, small-angle X-ray scattering, structural model, transcription, ChIP-Seq, chromatin immunoprecipitation–sequencing, DD, dimerization domain, HDX-MS, hydrogen-deuterium exchange mass spectrometry, MD, molecular dynamics, NTD, N-terminal domain, RHD, Rel-homology domain, SAXS, small-angle X-ray scattering, SEC, size-exclusion chromatography, TAD, transcription activation domain

## Abstract

Many transcription factors contain intrinsically disordered transcription activation domains (TADs), which mediate interactions with coactivators to activate transcription. Historically, DNA-binding domains and TADs have been considered as modular units, but recent studies have shown that TADs can influence DNA binding. Whether these results can be generalized to more TADs is not clear. Here, we biophysically characterized the NFκB p50/RelA heterodimer including the RelA TAD and investigated the TAD’s influence on NFκB–DNA interactions. In solution, we show the RelA TAD is disordered but compact, with helical tendency in two regions that interact with coactivators. We determined that the presence of the TAD increased the stoichiometry of NFκB–DNA complexes containing promoter DNA sequences with tandem κB recognition motifs by promoting the binding of NFκB dimers in excess of the number of κB sites. In addition, we measured the binding affinity of p50/RelA for DNA containing tandem κB sites and single κB sites. While the presence of the TAD enhanced the binding affinity of p50/RelA for all κB sequences tested, it also increased the affinity for nonspecific DNA sequences by over 10-fold, leading to an overall decrease in specificity for κB DNA sequences. In contrast, previous studies have generally reported that TADs decrease DNA-binding affinity and increase sequence specificity. Our results reveal a novel function of the RelA TAD in promoting binding to nonconsensus DNA, which sheds light on previous observations of extensive nonconsensus DNA binding by NFκB *in vivo* in response to strong inflammatory signals.

Precise control of gene activation and repression is mediated by both protein–protein and protein–DNA interactions involving transcription factor proteins that bind to specific DNA sequences in regulatory regions of genes and control the rate of transcription of mRNA (1). Eukaryotic transcription factors that activate genes often contain a transcription activation domain (TAD), which is a variable domain that binds transcription coactivators, ultimately recruiting the transcription preinitiation complex and RNA polymerase to initiate transcription (2, 3). TADs tend to be intrinsically disordered and enriched in aromatic and acidic amino acids (4, 5, 6).

Traditionally, transcription factors were considered modular units with separable DNA binding and transcription activation functions. For this reason, measurements of DNA-binding affinity and specificity *in vitro* have often used isolated DNA-binding domains. However, emerging work has shed light on different ways in which TADS and other disordered regions outside the DNA-binding domain can influence transcription factor–DNA interactions. In the examples studied so far, the presence of the TAD generally resulted in a decrease in DNA-binding affinity (7, 8, 9, 10) and/or an increase in DNA-binding specificity (11, 12, 13, 14). Given the dearth of quantitative studies of full-length transcription factors *in vitro*, it is unclear whether modulation of DNA-binding affinity and/or specificity by disordered domains is a general phenomenon.

Here, we characterize the transcription factor RelA (p65) in its full-length form, including its 230-residue TAD (Fig. 1*A*). RelA is a member of the NFκB family of transcription factors, which regulates at least 600 genes involved in processes including inflammation, immune response, differentiation, and cell survival (15, 16). The NFκB family consists of five proteins, which can form both homodimers and heterodimers (17). The most abundant NFκB dimer is the p50/RelA heterodimer, which is the focus of our work here. Both p50 and RelA contain a DNA-binding Rel-homology domain (RHD), which consists of an N-terminal domain (NTD) and a dimerization domain (DD) connected by a short, flexible linker. The inhibitor protein IκBα holds p50/RelA heterodimers in the cytoplasm under resting conditions. In response to stimuli, IκBα is ubiquitinated and degraded, freeing p50/RelA heterodimers to translocate to the nucleus, bind DNA sequences, and regulate transcription (18). To terminate the signal, newly synthesized IκBα binds to DNA-bound NFκB and facilitates its dissociation from DNA in a process we have called molecular stripping (19, 20). A mutant form of IκBα which was defective in stripping NFκB from DNA *in vitro* showed much slower nuclear export of NFκB in cells, demonstrating the physiological function of IκBα-mediated stripping (21).

**Figure 1:**
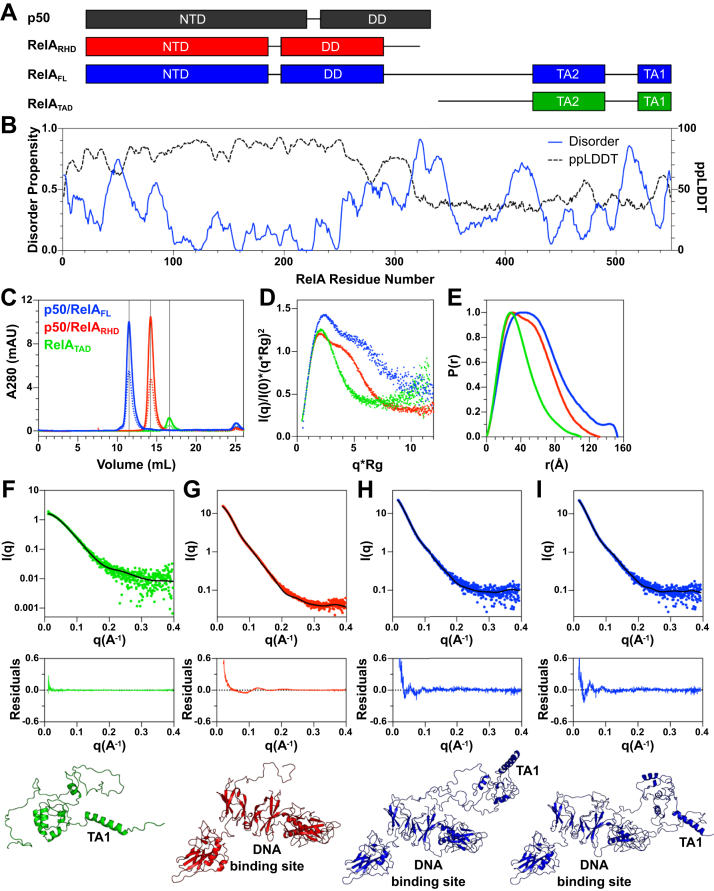
**Characterization of NFκB constructs used in this work.***A*, NFκB subunits p50 and RelA contain well-folded N-terminal domains (NTDs) and dimerization domains (DDs) that make up the Rel-homology domain (RHD). Additionally, RelA contains an intrinsically disordered transcription activation domain (TAD), which contains two regions important for protein–protein interactions, TA1 and TA2. *B*, the disorder propensity of full-length RelA (*blue*, solid) and the predicted AlphaFold2 pLDDT score (*black*, *dashed*) were predicted using MetaPredict. The TAD (residues 320–549) is largely predicted to be disordered except for two short stretches corresponding to the TA1 and TA2 motifs. Note that the graph residue numbers are aligned with the RelA_FL_ (residues 19–549) cartoon in panel (*A*). *C*, the three protein constructs used in this work, p50/RelA_FL_ (*blue*), p50/RelA_RHD_ (*red*), and RelA_TAD_ (*green*) were analyzed by analytical SEC. *Solid lines* represent 10 μM p50/RelA_FL_ and p50/RelA_RHD_ and 20 μM RelA_TAD_. *Dashed lines* represent 5 μM p50/RelA_FL_ and p50/RelA_RHD_ and 10 μM RelA_TAD_. *D*, SAXS Kratky plots p50/RelA_FL_ (*blue*), p50/RelA_RHD_ (*red*), and RelA_TAD_ (*green*). *E*, pairwise distance distribution for p50/RelA_FL_ (*blue*), p50/RelA_RHD_ (*red*), and RelA_TAD_ (*green*). The R_g_ and D_max_ values of each construct based on SAXS data analysis are in Table 1. *F*, a model of RelA_TAD_ consistent with the SAXS data was generated using the AWSEM and BilboMD. *G*, a model of p50/RelA_RHD_ consistent with the SAXS data was generated using BilboMD. *H* and *I*, two of the models of p50/RelA_FL_ that were generated using FoXSDock and BilboMD represent two possible conformations of this protein that agree well with the SAXS data. SAXS, small-angle X-ray scattering; SEC, size-exclusion chromatography.

Multiple crystal structures have been solved of the p50/RelA heterodimer RHD bound to DNA (22, 23, 24, 25). In all structures, the NTDs of both NFκB subunits contact nucleotide bases within the major groove, whereas the DDs bind each other and interact with the DNA backbone. No structures of NFκB dimers containing both NTDs have been solved in the absence of DNA, and molecular dynamics (MD) simulations and single-molecule FRET experiments have demonstrated that the two NTDs in p50/RelA are highly dynamic and can swing apart and create a larger cavity in both the absence and presence of DNA (26, 27). The RelA TAD is necessary for efficient transcription activation of nearly all target genes (28, 29). However, previous structural and biophysical studies have characterized short segments of this domain in isolation, and its behavior within the full-length p50/RelA heterodimer is uncharacterized (30, 31).

The p50/RelA heterodimer binds to κB DNA sites that contain the consensus sequence GGGRNNYYCC, where R denotes a purine, N denotes any nucleotide, and Y denotes a pyrimidine (32). However, high-throughput *in vitro* analysis has shown that it can bind with similar affinity to some sequences that differ from the consensus, including sequences that contain only a half-site (33, 34). An important open question in NFκB signaling is how NFκB dimers achieve signaling specificity upon entering the nucleus. Across multiple experiments, one third to one half of strong chromatin immunoprecipitation–sequencing (ChIP-Seq) peaks bound by RelA did not overlap with a consensus κB motif (35, 36, 37, 38). The number of specific κB sites in the human genome was estimated to be ∼10^3^ to 10^4^, whereas the number of RelA molecules that flood the nucleus in response to a strong activating signal is estimated to be ∼10^5^ (18, 39, 40, 41). Many questions remain unanswered regarding NFκB specificity, including how *in vivo* results involving full-length RelA relate to *in vitro* experiments lacking the TAD.

Most genes activated by NFκB transcription factors contain multiple κB sites in their promoter and/or enhancer regions. Tandem DNA-binding sites are often associated with cooperative binding and efficient transcription activation (42, 43, 44), but the effect of tandem κB sites is not well studied in the context of NFκB-DNA interactions. On a cellular level, many genes activated by NFκB that contain multiple κB sites show a graded response to stimuli, and the transcriptional output by NFκB best matches a model without cooperativity between sites (45). The only DNA sequence containing tandem κB sites that has been studied extensively *in vitro* is the HIV LTR promoter sequence, which has two κB sites separated by four nucleotides. Qualitative experiments have suggested that two p50/RelA heterodimers bind with negative cooperativity due to the steric clash generated by the short intervening sequence between the two κB sites (23, 46). Binding of NFκB to tandem κB sites has not been studied quantitatively *in vitro*, and no experiments have been performed with an NFκB construct including the RelA TAD.

In this work, we characterize the structural propensity of the RelA TAD alone and in the context of the full-length p50/RelA heterodimer using small-angle X-ray scattering (SAXS), computational modeling, and hydrogen-deuterium exchange mass spectrometry (HDX-MS). We then investigate whether and how the presence of the TAD influences DNA binding stoichiometry, affinity, specificity, and cooperativity to better understand how the full-length p50/RelA heterodimer engages DNA. We find that the RelA TAD is structurally compact but intrinsically disordered both alone and in the context of the p50/RelA heterodimer. Inclusion of the RelA TAD increases the stoichiometry of p50/RelA binding to DNA sequences containing tandem κB sites by promoting binding of p50/RelA dimers in excess of the number of κB sites. It enhances the binding of p50/RelA to all DNA sequences tested, but this effect is more pronounced for DNA sequences that do not match the consensus. Together, these results support a model in which full-length NFκB preferentially binds consensus DNA sequences but also recognizes nonspecific DNA, particularly when present in stoichiometric excess as occurs in the nucleus following inflammatory stimulation. This novel role of the RelA TAD helps explain previous *in vivo* observations of widespread nonconsensus DNA binding by RelA.

## Results

### Solution characterization of p50/RelA_FL_, p50/RelA_RHD_, and RelA_TAD_

We expressed and purified p50_39-350_/RelA_19-549_ (hereafter referred to as p50/RelA_FL_), p50_39-350_/RelA_19-321_ (hereafter referred to as p50/RelA_RHD_), and RelA_340-549_ (hereafter referred to as RelA_TAD_) protein constructs recombinantly from *Escherichia coli* and characterized the biophysical properties of the RelA TAD alone and within the full-length p50/RelA heterodimer (Fig. 1*A*). To gain a better understanding of the disordered tendency of the RelA TAD, we used the Metapredict web server (47). Metapredict provides two predicted parameters for a given protein sequence: the disorder propensity, which is designed to reproduce consensus disorder scores from other disorder predictors, and the predicted pLDDT (predicted local distance difference test) (ppLDDT), which is a prediction of the AlphaFold2 pLDDT score. The AlphaFold2 pLDDT score is a residue level confidence metric representing the probability that an AlphaFold structural prediction for a local region will match an experimentally determined structure (48). Low pLDDT scores have been shown to predict disordered regions with high accuracy (49) and provide a useful complement to traditional disorder predictors. The disorder probability scores are low and the ppLDDT scores are high for the first 300 residues of RelA, reflecting the ordered nature of the RHD (Fig. 1*B*). For the RelA TAD (residues 320–549), the ppLDDT score is below 50% and the disorder propensity score is high with the exception of short stretches including residues ∼430 to 500 and ∼530 to 545. These regions correspond to the TA2 and TA1 motifs, respectively, which have previously been shown to form alpha helices in complex with transcription coactivators (30, 31). These prediction results paint an overall picture of a largely disordered TAD with local regions of secondary structure propensity.

To confirm that the protein constructs used in this work were soluble and did not form higher order oligomers, we analyzed the protein samples at two concentrations by analytical size-exclusion chromatography (SEC). Each protein eluted as a single symmetric peak from SEC at the expected volume for the mass of the protein construct, and there was no concentration dependence on elution volume (Fig. 1*C*). This confirms that the proteins used in this study are soluble and do not form higher order oligomers at the concentrations used in the experiments presented here.

We used SAXS to gain insight into the molecular shapes of each protein construct in solution. Kratky analysis of RelA_TAD_ revealed that it adopts a compact structure containing disordered regions, indicated by a Gaussian peak at low q values with a plateau at high q values (Fig. 1*D*). Kratky analysis of p50/RelA_RHD_ and p50/RelA_FL_ showed both have a multidomain architecture (Fig. 1*D*), as expected due to the presence of the multidomain RHD. Additionally, the Kratky plot for p50/RelA_FL_ plateaus at high q values, indicating the presence of disordered residues. Average radii of gyration (R_g_) of RelA_TAD_, p50/RelA_RHD_, and p50/RelA_FL_ were determined by Guinier approximation to be 27.3 ± 0.2 Å, 37.1 ± 0.2 Å, and 46.2 ± 1.3 Å, respectively (Table 1). Notably, the R_g_ value of 27.3 ± 0.2 Å for RelA_TAD_ is intermediate between the expected R_g_ of a folded protein containing 218 amino acids (∼18–20 Å) and the R_g_ of an excluded volume polymer of the same size (∼48 Å) (50, 51). The P(r) curves of RelA_TAD_, p50/RelA_RHD_, and p50/RelA_FL_ showed D_max_ of 82 Å, 110 Å, and 153 Å respectively (Fig. 1*E* and Table 1). Overall, these results are consistent with the RelA TAD having a compact conformation while retaining a high degree of disorder both when expressed on its own and within p50/RelA_FL_.

**Table 1:** SAXS analysis parameters for p50/RelA_FL_, p50/RelA_RHD_ and RelA_TA__D_

SAXS parameter	RelA_TAD_	p50/RelA_RHD_	p50/RelA_FL_
R_g_ (Å), Guinier analysis	27.3 ± 0.2	37.1 ± 0.2	46.2 ± 1.3
R_g_ (Å), P(r) analysis	27.2 ± 0.1	37.1 ± 0.2	46.3 ± 0.2
D_max_ (Å), P(r) analysis	82	110	153

To aid in visualization of possible structural conformations, we computationally generated structural models of each construct that were consistent with the SAXS data (Fig. 1,*F*–*I* and Table 2). Given the dynamic and disordered nature of these protein constructs, our goal was not to produce a single definitive structure but to present models that depict possible configurations of the proteins to enable visualization of their degree of compactness and structural propensity. All models generated had Χ^2^ values less than 1.5 when compared to the SAXS data and had similar radii of gyration to those determined *via* SAXS, indicating they are in good agreement with our experimental results (Tables 1 and 2). Models of RelA_TAD_ were initially predicted using the AWSEM code (52) and refined using BilboMD and MultiFoXS to achieve the best agreement with the SAXS data (Fig. 1*F* and Table 2). The best-fitting representative model has a relatively compact conformation that is mostly unstructured but has some helical content in the TA1 and TA2 regions. We expect that the RelA TAD adopts many different conformations, including both more compact and more extended states within the structural ensemble, and our model represents one possible conformation with the average R_g_.

**Table 2:** SAXS model parameters for p50/RelA_FL_, p50/RelA_RHD_ and RelA_TAD_

Model parameter	RelA_TAD_	p50/RelA_RHD_	p50/RelA_FL_ Model1/Model2
Χ^2^	1.1	1.35	1.44/1.43
c_1_	0.99	1.04	1.04/1.05
c_2_	3.40	0.30	−0.56/−0.24
R_g_	26.7	37.0	46.4/45.2

A model of p50/RelA_RHD_ consistent with the SAXS data was generated by refining a structure that was generated by removing the DNA from Protein Data Bank code 1LE5 (24) and running MD simulations for 400 ns. In this structure, the p50 NTD is further away from the RelA NTD, generating a wider DNA-binding cavity (26). This structure was further refined using BilboMD to better fit the SAXS data (Fig. 1*G* and Table 2). The distance between the two NTDs in this model is wider than in published crystal structures of the NFκB–DNA complex (24) but consistent with single-molecule FRET studies, showing the NTDs are dynamic relative to each other and can adopt open conformations in solution (27).

Models of p50/RelA_FL_ were generated by docking the model of RelA_TAD_ onto a model of p50/RelA_RHD_ using FoXSDock. Modeller and BilboMD were used to refine the models that best fit the SAXS data. Excellent Χ^2^ values were obtained for two different models in which the TA1 helix extends into solution and the TA2 region is also exposed (Fig. 1,*H* and *I* and Table 2). The long proline-rich sequence that connects the RHD to the TA2 region adopted several different conformations. These models were neither fully extended nor fully compact, matching the intermediate R_g_ measured by SAXS. We believe the structures represent a subset of the possible conformations of the TAD, which remains highly dynamic in the context of p50/RelA_FL_. Overall, these results match the findings for other acidic, hydrophobic-rich TADs, in which hydrophobic and aromatic amino acids important for protein-protein interactions remain solvent exposed within disordered, negatively charged regions (5, 6, 53).

### HDX-MS analysis of p50/RelA_FL_ and p50/RelA_RHD_

**Figure 2:**
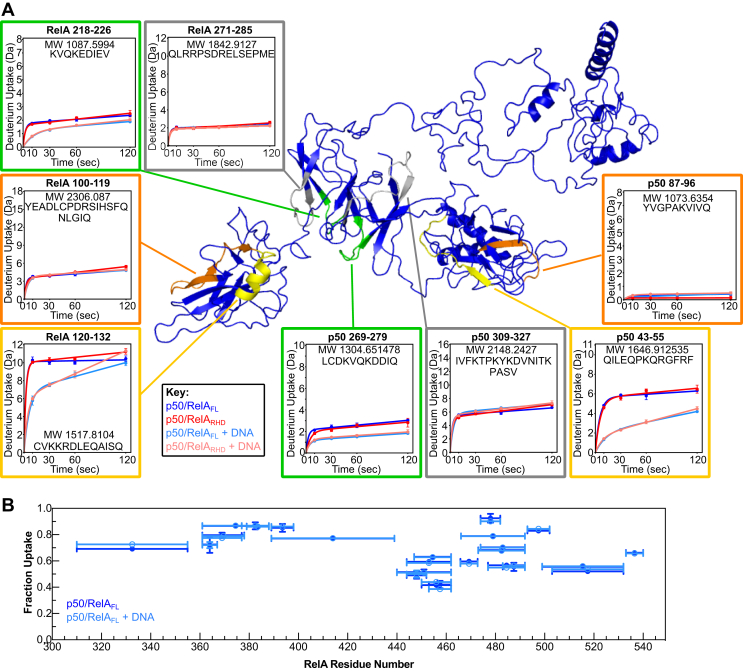
**Hydrogen-deuterium exchange analysis of NFκB constructs alone and bound to DNA.***A*, the constructs p50/RelA_FL_ and p50/RelA_RHD_ alone and bound to a DNA hairpin containing the HIV-LTR κB sequence were analyzed using hydrogen-deuterium exchange mass spectrometry (HDX-MS). Representative deuterium uptake plots are shown mapped to the structural model of p50/RelA_FL_. Peptides in the DNA-binding cavity showed reduced deuterium incorporation in the presence of DNA (*green* and *yellow* peptides highlighted above, representing the DBD and NTD respectively), whereas peptides outside the cavity did not show significant changes in deuterium incorporation in the presence of DNA (*gray* and *orange* peptides highlighted above, representing the DBD and NTD respectively). In general, there were no significant differences in deuterium incorporation when comparing p50/RelA_FL_*versus* p50/RelA_RHD_ alone or bound to DNA. *B*, the deuterium uptake of peptides in the RelA TAD in the presence (*light blue*) and absence (*blue*) of DNA. Horizontal bars represent the span of residues in each peptide, and the fraction uptake represents the number of deuterons incorporated after 10 s relative to the number of exchangeable hydrogen atoms in the peptide. Vertical error bars represent the SD of percent uptake based on three technical replicates. NTD, N-terminal domain; TAD, transcription activation domain.

To gain higher resolution information about the structure and dynamic properties of p50/RelA_FL_ in solution, we conducted HDX-MS experiments of p50/RelA_FL_ and p50/RelA_RHD_ alone and of each construct bound to a DNA hairpin containing the HIV-LTR κB sequence. We monitored amide hydrogen exchange following 10, 30, 60, and 120 s incubations in a deuterium-based buffer, which reports on protein dynamics in the microsecond to millisecond time regime (54). Overall, the results are consistent with the SAXS and modeling data presented before. The models of p50/RelA_FL_ and p50/RelA_RHD_ that best fit the SAXS data did not show stable interactions between the TAD and the RHD. Consistent with these models, no significant differences in deuterium uptake were observed within the RHD whether or not the TAD was present (Fig. 2*A*). Thus, the RelA TAD does not appear to form long-lived contacts with the RHD that alter the rate of deuterium uptake by this domain. For both protein constructs, regions contacting the DNA-binding cavity in both the NTDs and DDs of p50 and RelA incorporated significantly less deuterium in the presence of DNA (Fig. 2*A*, *green* and *yellow* peptides). Regions outside the DNA-binding cavity did not show significant changes in deuterium incorporation upon DNA binding (Fig. 2*A*, *gray* and *orange* peptides). Again, we did not detect significant differences in deuterium incorporation between p50/RelA_FL_ and p50/RelA_RHD_ in the DNA-bound state. Based on these results, we can conclude that the RelA TAD does not form long-lived contacts with the DNA-bound RHD in a manner that alters the rate of deuterium incorporation by the RHD (Fig. 2*A*).

The RelA TAD showed high levels of deuterium incorporation throughout its sequence, consistent with its disordered, solvent-accessible nature. Most of the TAD had exchanged 70% or more by the 10 s time point, with the exception only in the TA1 and TA2 regions (Fig. 2*B*). Peptides from the regions spanning from residues 440 to 473, 483 to 492, and 499 to 540 showed less than 70% deuterium incorporation at this time point, perhaps reflecting their ability to form helical secondary structure as predicted by the AWSEM simulations. Notably, we did not detect differences in deuterium incorporation in peptides from the RelA TAD when comparing the DNA bound and unbound states (Fig. 2*B*).

### Binding stoichiometry analysis of NFκB constructs to DNA sequences with tandem sites

**Figure 3:**
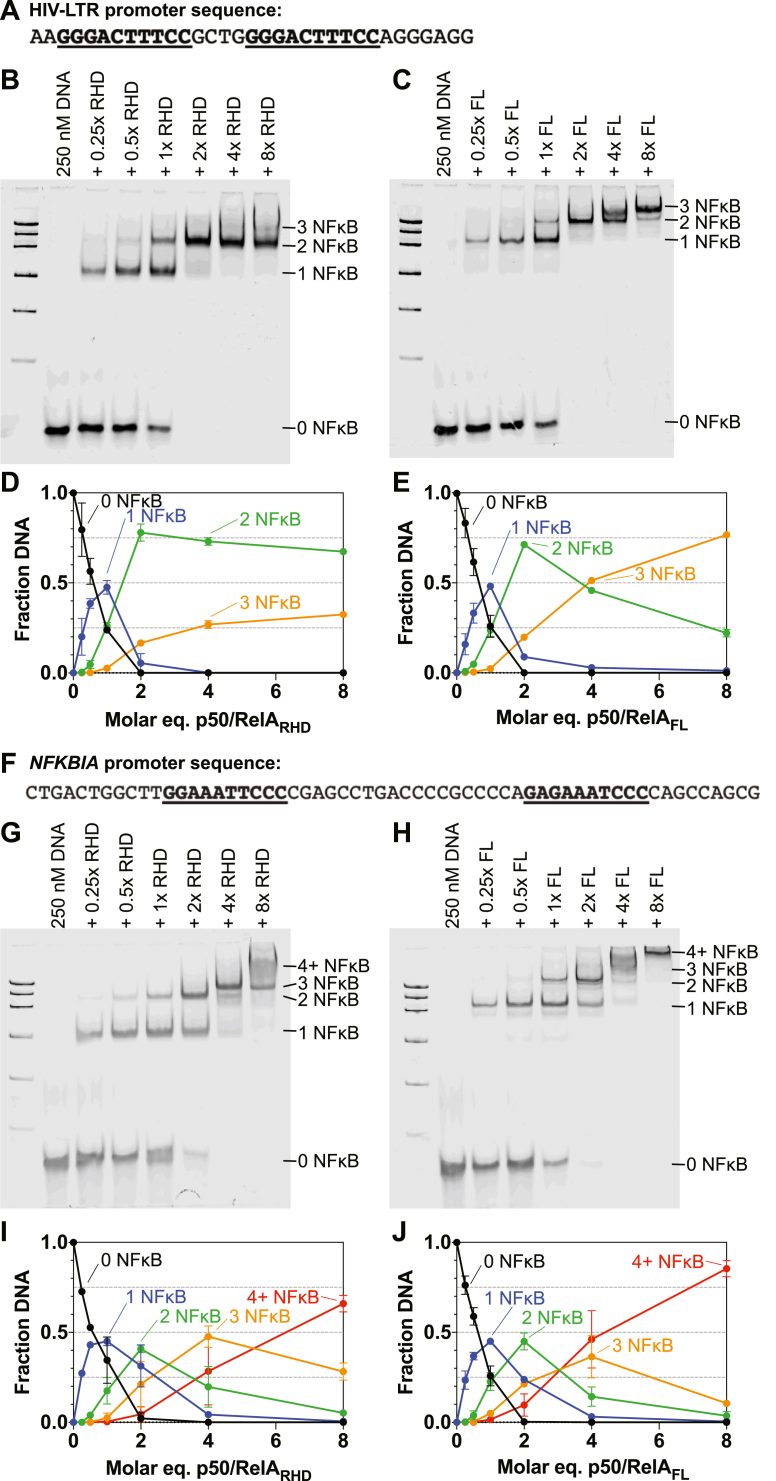
**EMSA analysis of binding stoichiometries of p50/RelA**_**FL**_**and p50/RelA**_**RHD**_**to tandem DNA sequences.***A*, a 33 bp segment of the HIV LTR promoter sequence was used in these experiments. *B*, binding of p50/RelA_RHD_ (0, 62.5, 125, 250, 500, 1000, and 2000 nM) to the HIV LTR promoter DNA (250 nM) was detected using EMSA. Bands are visible corresponding to free DNA and DNA bound by 1, 2, or 3 p50/RelA_RHD_ dimers. *C*, binding of varying concentrations of p50/RelA_FL_ to the HIV LTR promoter DNA (250 nM) was detected using EMSA. *D* and *E*, the intensities of the bands in the EMSA gels detecting binding of p50/RelA_RHD_ (*D*) and p50/RelA_FL_ (*E*) to the HIV LTR DNA were quantified using ImageJ and plotted as a function of NFκB concentration. Data points represent the mean and SD of two biological replicates. *F*, a 59 bp segment of the *NFKBIA* promoter sequence was used in these experiments. *G*, binding of p50/RelA_RHD_ (0, 62.5, 125, 250, 500, 1000, and 2000 nM) to the *NFKBIA* promoter sequence (250 nM) was analyzed using EMSA. The smear present near the top of the gel at higher p50/RelA_RHD_ concentrations likely represents DNA bound by four or more p50/RelA_RHD_ dimers. *H*, binding of p50/RelA_FL_ to the *NFKBIA* promoter sequence was analyzed using EMSA. *I* and *J*, the intensities of the bands in the EMSA gels detecting binding of p50/RelA_RHD_ (*I*) and p50/RelA_FL_ (*J*) to the *NFKBIA* DNA were quantified using ImageJ and plotted as a function of NFκB concentration. Data points represent the mean and SD of two independent biological replicates.

To better understand the role of the RelA TAD in binding to DNA, we used EMSAs to investigate the binding of p50/RelA_FL_ and p50/RelA_RHD_ to native DNA promoter sequences containing tandem κB sites. We investigated two tandem sequences: the HIV LTR promoter, which contains two identical κB sites separated by 4 bp (Figs. 3*A* and S1), and the *NFKBIA* promoter, which contains nonidentical κB sites separated by 19 bp (Figs. 3*F* and S1) (23, 55). Notably, the two κB sites in the *NFKBIA* promoter are quite different from each other, as the second site is a half-site. The HIV LTR promoter has been studied extensively, and a crystal structure of it bound by two p50/RelA_RHD_ dimers has been solved (46). While previous studies have suggested that p50/RelA_RHD_ binds to these sites with negative cooperativity (23, 46), the binding affinities driving this association have not been determined quantitatively and binding has not been investigated in the presence of the RelA TAD. Binding of two NFκB dimers to the *NFKBIA* promoter sequence has not been characterized *in vitro* to our knowledge.

Varying concentrations of NFκB were incubated with 250 nM dsDNA segments, then run on a native polyacrylamide gel and stained to observe the distribution of DNA species. Notably, the concentrations used in these experiments are well above the *K*_*d*_ values previously determined for specific NFκB-DNA interactions in the absence of the RelA TAD (56) and reported later in this article for the p50/RelA_FL_. For the HIV LTR sequence at low concentrations of NFκB, the EMSA results suggested a lack of binding cooperativity and a similar binding affinity for each κB site. For both p50/RelA_FL_ and p50/RelA_RHD_, the ratio of unbound DNA to DNA bound by a single NFκB dimer to DNA bound by two NFκB dimers is 1:2:1 when NFκB is present at an equimolar concentration to the DNA (Fig. 3, *B*–*E*). This is the expected distribution of species if NFκB dimers bind the two κB sites in the DNA sequence with the same affinity and without positive or negative cooperativity.

When present in stoichiometric excess, NFκB formed higher order complexes with the HIV-LTR DNA containing up to three NFκB dimers. This effect was observed for both p50/RelA_FL_ and p50/RelA_RHD_ when present in 8-fold excess but was much more pronounced for p50/RelA_FL_. Binding of more than two NFκB dimers to this DNA requires binding to nonconsensus sequences. The DNA used in these experiments was 33 bp long, sufficiently long to accommodate up to three NFκB dimers, assuming each dimer requires a 10 bp segment to bind efficiently. However, the DNA does not contain a 10 bp stretch of nonspecific DNA that does not overlap with a κB site. Therefore, it could not accommodate a third NFκB dimer without displacing a dimer bound specifically to a κB site.

The results obtained using the *NFKBIA* promoter DNA are similar in pattern to the HIV-LTR results but more complicated due to the longer length of the *NFKBIA* promoter sequence (59 bp). Individual bands can be distinguished for free DNA and DNA bound by 1, 2, or 3 NFκB molecules, and higher order complexes appear as a smear on the gel. As observed for the HIV-LTR DNA, p50/RelA_FL_ displays a greater propensity toward higher order complex formation than p50/RelA_RHD_ (Fig. 3, *G*–*J*). Importantly, the highest concentration of NFκB used in this experiment (2 μM) and the ratio of NFκB dimers to specific κB sites (4:1), are not unlike the condition in the nucleus, which can be flooded by ∼10^5^ NFκB dimers that recognize ∼10^4^ specific κB sites in response to strong activating signals (41). We estimate the nuclear concentration of p50/RelA heterodimers to be in the 2 to 4 μM range under such conditions (see Discussion).

**Figure 4:**
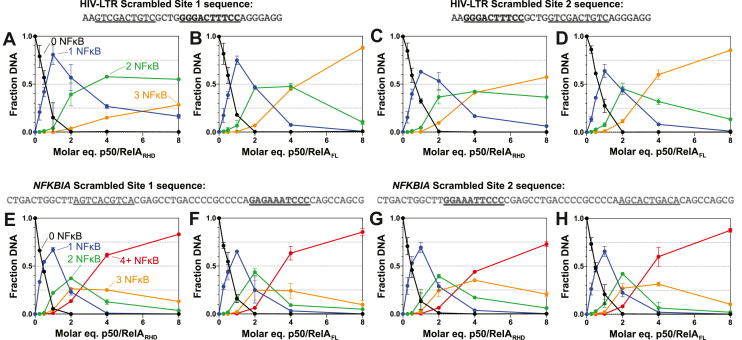
**EMSA analysis of binding stoichiometries of p50/RelA**_**FL**_**and p50/RelA**_**RHD**_**to mutated DNA sequences.***A* and *B*, binding of p50/RelA_RHD_ and p50/RelA_FL_ to the HIV LTR sequence in which the first κB site is scrambled was detected using EMSA. Varying concentrations of p50/RelA_RHD_ and p50/RelA_FL_ were mixed with 250 nM HIV-LTR DNA in which the first κB site is scrambled and analyzed using EMSAs. The fraction of DNA bound by 0, 1, 2, or 3 p50/RelA dimers was quantified using ImageJ and plotted as a function of p50/RelA concentration. *C* and *D*, when the second κB site in the HIV-LTR promoter DNA segment is scrambled, 21 bp of nonspecific DNA are present following the first κB site. Varying concentrations of p50/RelA_RHD_ and p50/RelA_FL_ were mixed with 250 nM HIV-LTR DNA in which the second κB site is scrambled and analyzed using EMSAs. Compared to WT HIV-LTR DNA and HIV-LTR DNA in which the first site is scrambled, both p50/RelA_RHD_ and p50/RelA_FL_ formed more complexes in which 3 p50/RelA dimers were bound to the DNA. *E* and *F*, similar experiments were conducted using a DNA segment consisting of the *NFKBIA* promoter sequence with the first κB site scrambled. *G* and *H*, binding of p50/RelA_RHD_ and p50/RelA_FL_ to the *NFKBIA* sequence with the second κB site scrambled was also monitored using EMSAs. Overall, scrambling either site in the *NFKBIA* promoter led to an increase in higher order complex formation at high concentrations of p50/RelA_RHD_ and p50/RelA_FL_. All data points represent the mean and SD of two independent experiments.

We tested the effect of mutating one of the κB sites in the HIV-LTR and *NFKBIA* sequences while leaving the other intact (Figs. 4, and S1). For HIV-LTR DNA, we found that mutating either the first or the second site resulted in an increase in DNA bound by a single NFκB dimer and a decrease in DNA bound by two NFκB dimers relative to the WT DNA sequences when NFκB was present in substoichiometric amounts (Fig. 4, *A*–*D*). This result is expected, given that the scrambled DNA sequences contain a single κB site instead of two. Somewhat counterintuitively, at high NFκB concentrations, the mutation of one κB site did not diminish NFκB binding and for some sequences actually increased the number of NFκB dimers bound. The amount of DNA bound by three of NFκB dimers when the first site was scrambled was similar to the results obtained using the WT DNA (Figs. 3, *D*, *E*, 4, *A* and *B*), but we observed significantly more triply bound DNA when the second site was scrambled (Fig. 4, *C* and *D*). This result held for both p50/RelA_RHD_ and p50/RelA_FL_, although again p50/RelA_FL_ was more prone to higher order complex formation than p50/RelA_RHD_. Notably, scrambling the second site results in 21 sequential base pairs of nonspecific DNA, which can accommodate two NFκB dimers without displacing the dimer bound to the specific κB site. By contrast, scrambling the first κB site results in only 16 sequential base pairs of nonspecific DNA, and in order to bind 3 NFκB dimers, this sequence must bind all three nonspecifically.

The results obtained using the *NFKBIA* promoter sequence with one site scrambled are generally consistent with the results using the HIV-LTR. For each scrambled *NFKBIA* sequence, DNA bound by a single NFκB dimer is more prevalent at low concentrations than DNA bound by two NFκB dimers relative to the WT DNA, reflecting the presence of only one κB site (Fig. 4, *E*–*H*). At higher NFκB concentrations, more complexes formed with three or more NFκB molecules bound to the scrambled DNA relative to the WT DNA. Again, p50/RelA_FL_ was more prone to higher order complex formation than p50/RelA_RHD_.

In summary, both p50/RelA_FL_ and p50/RelA_RHD_ can form complexes with DNA in which the number of proteins bound to a strand of DNA exceeds the number of κB sites. For all sequences tested, p50/RelA_FL_ is more prone to formation of these higher order complexes than p50/RelA_RHD_. Higher order complexes are more likely to form when stretches of 10 bp or longer are accessible without displacement of NFκB dimers from specific κB sites.

### Consequences of specific and nonspecific DNA interactions

**Figure 5:**
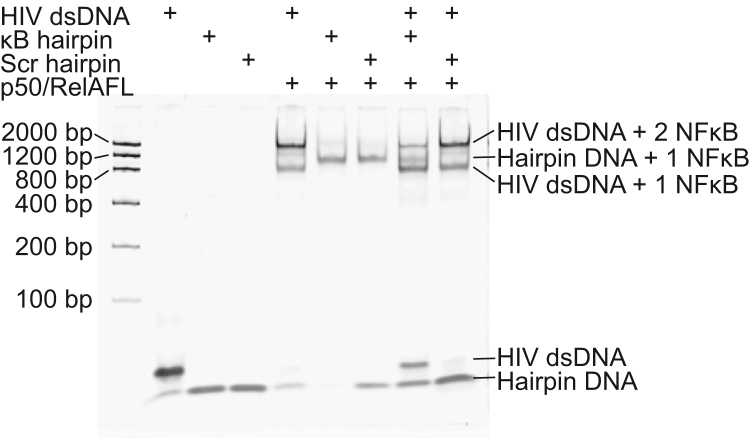
**Competition between specific and nonspecific DNA sequences for p50/RelA**_**FL**_**binding.** 250 nM double-stranded HIV-LTR DNA was incubated with 500 nM p50/RelA_FL_, and 250 nM hairpin DNA containing either the HIV-LTR κB sequence or a scrambled sequence was added to the sample. The κB hairpin was able to efficiently compete with the HIV LTR dsDNA for p50/RelA_FL_ binding (comparing lanes 5 & 8), whereas the scrambled DNA hairpin was unable to compete with the HIV LTR dsDNA for p50/RelA_FL_ binding (comparing lanes 5 & 9).

To better understand how p50/RelA_FL_ might distinguish between specific and nonspecific DNA sequences within the nucleus, we conducted EMSA experiments in which we added specific or non-specific hairpin DNA to compete with binding to the HIV-LTR promoter DNA containing tandem κB sites (Fig. 5). Without competitor, most of the HIV-LTR DNA is bound by two p50/RelA_FL_ dimers when p50/RelA_FL_ is present at a 2:1 M ratio. However, when a DNA hairpin containing the HIV κB sequence is included, it efficiently competes with the HIV-LTR tandem DNA for p50/RelA_FL_ binding. The EMSA band containing HIV-LTR DNA bound by two p50/RelA_FL_ dimers decreases in intensity in the presence of the HIV κB hairpin, while the bands for singly bound HIV-LTR and free HIV-LTR DNA increase in intensity. A band corresponding to hairpin DNA bound by NFκB is also present.

By contrast, addition of a hairpin with a scrambled DNA sequence does not appreciably change the binding pattern of p50/RelA_FL_ to the HIV-LTR DNA. The p50/RelA_FL_-bound DNA bands match the pattern and intensity of the sample without a competitor hairpin, and a band of free HIV-LTR DNA does not appear (Fig. 5). Therefore, the addition of excess nonspecific DNA does not efficiently compete with the specific tandem DNA for binding by NFκB.

We conducted the same experiment using a 59 bp dsDNA sequence (the *NFKBIA* promoter sequence with both κB sites scrambled) to test whether a longer stretch of nonspecific DNA might be a better competitor for NFκB binding. We generally observed higher affinity binding to longer, dsDNA segments than to hairpins in our fluorescence anisotropy assays, so we wanted to test whether excess dsDNA could compete with the specific DNA for NFκB binding. Although these results were harder to interpret due to overlap between NFκB-bound DNA bands, the overall result was the same as with the hairpin DNA. A band of free HIV LTR tandem DNA appeared when the HIV hairpin DNA was included as a competitor but not when the nonspecific dsDNA was present (Fig. S2).

Based on these results, we believe that despite the lower DNA-binding specificity of p50/RelA_FL_ compared to a construct lacking the TAD, it retains a clear preference for specific κB sites relative to nonspecific DNA. Under nucleus-like conditions in which there is an excess of nonspecific DNA, we predict it would preferentially bind specific sites. Nevertheless, we anticipate that nonspecific DNA interactions may play important roles in NFκB signaling, particularly when NFκB molecules are present in stoichiometric excess relative to the number of κB sites, as is the case during induction of the NFκB signaling system by stressors such as TNF.

### Quantitative determination of DNA binding affinity by p50/RelA_FL_

**Figure 6:**
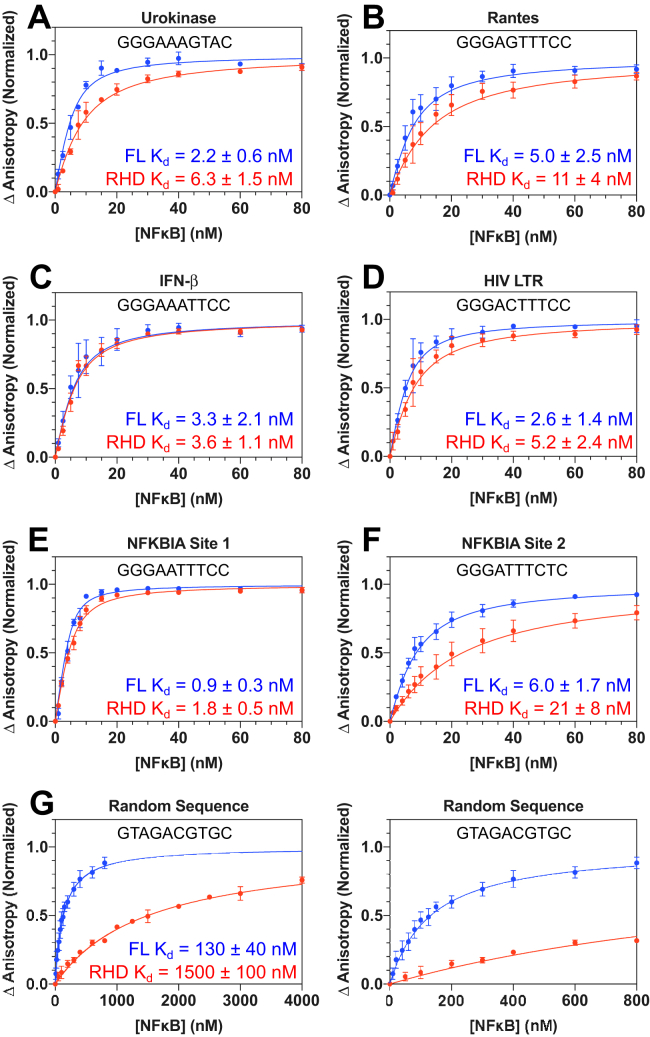
**Binding affinities of p50/RelA**_**FL**_**and p50/RelA**_**RHD**_**for hairpin DNA.***A*–*G*, DNA hairpins were labeled on the 5′ ends with fluorescein, and 5 nM DNA was incubated with varying concentrations of p50/RelA_FL_ (*blue*) and p50/RelA_RHD_ (*red*) to determine binding affinity *via* fluorescence anisotropy. Note that the same data are graphed twice in panel (*G*) to enable clear visualization of both binding curves. Data points represent the mean and SEM of three independent experiments, and the reported *K*_*d*_ values are the mean and SEM of the *K*_*d*_ values derived from each of the experiments individually. Lines represent the expected binding curve based on the mean *K*_*d*_ values.

We used fluorescence anisotropy to determine the equilibrium binding affinities of p50/RelA_FL_ and p50/RelA_RHD_ for several different naturally occurring DNA sequences to gain a more quantitative understanding of how the RelA TAD influences DNA binding. First, DNA hairpins containing the sequences of six κB sites including the *NFKBIA* promoter, the HIV LTR promoter, the urokinase promoter, the *IFN-β* enhancer, and the *RANTES* promoter were fluorescently labeled on their 5′ ends, and the change in fluorescence anisotropy was monitored upon titration with NFκB constructs (Fig. 6, *A*–*F*). Of note, the urokinase and *NFKBIA* site 2 sequences are both half κB sites, while the others are full κB consensus sequences. In addition, a hairpin containing a random DNA sequence was included to measure binding affinity for nonspecific DNA (Fig. 6*G*).

In general, p50/RelA_FL_ bound to κB DNA sequences with *K*_*d*_ values between 1 and 6 nM, whereas p50/RelA_RHD_ bound with a range of *K*_*d*_ values between 2 and 20 nM. The p50/RelA_FL_ construct bound with average higher affinity to all the DNA sequences tested compared to p50/RelA_RHD_, although this was not statistically significant for all sequences. Most dramatically, p50/RelA_FL_ bound the random DNA hairpin with 10-fold higher affinity than p50/RelA_RHD_, 130 ± 40 nM *versus* 1500 ± 100 nM (*p* = 0.0002). For the half-site urokinase promoter, p50/RelA_FL_ bound with 3-fold higher affinity than p50/RelA_RHD_ with *K*_*d*_ values of 2.2 ± 0.6 nM *versus* 6.3 ± 1.5 nM, respectively (*p* = 0.037). The other sequences did not have statistically significant differences in binding affinity between p50/RelA_FL_ and p50/RelA_RHD_. The *p* value for the other half-site, NFKBIA, was 0.085, and the *p*-values for all the full κB sequences were greater than 0.1. Taken together, there is a trend in which the presence of the TAD causes greater binding enhancement for DNA sequences that differ from the full κB consensus sequence. This enhanced binding affinity for nonspecific DNA is consistent with the EMSA results showing a greater propensity for p50/RelA_FL_ to bind DNA in excess of the number of κB sites (Figs. 3 and 4).

We devised a method to detect binding to each site within the tandem κB sequences individually in order to quantitatively determine the binding affinities for each site within the longer sequence. When a fluorophore is conjugated to the 5′ end of the DNA in close proximity (within 3 bp) of a κB site, it becomes immobilized when an NFκB dimer binds to that site, leading to a change in fluorescence anisotropy (57). We found that the change in anisotropy is highly dependent on the distance between the fluorophore and the nearest κB binding site and drops off sharply at distances greater than 3 bp (Fig. S3, *A*–*C*). By creating dsDNA with 3 bp on either side of the tandem κB sites, we can detect binding to either site by monitoring fluorescence anisotropy of a fluorophore conjugated to the 5′ end of either the forward or reverse DNA strand. When the 5′ end of the forward strand is labeled, the change in anisotropy reflects binding to the first κB site, whereas when the 5′ end of the reverse strand is labeled, the change in anisotropy reflects binding to the second site. Under the conditions used in these experiments, we do not expect binding at the farther site to contribute to the change in anisotropy (Fig. S3, *C* and *D*). The results of the equilibrium binding titrations with labels on each strand were fit globally using MATLAB to determine the binding dissociation constants for each site.

**Figure 7:**
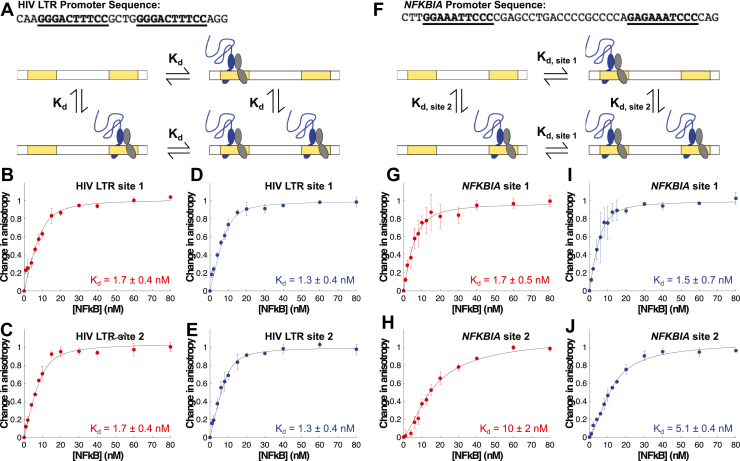
**Binding affinities of p50/RelA**_**FL**_**and p50/RelA**_**RHD**_**for κB sites within tandem DNA sequences.***A*, dsDNA containing the sequence of the HIV LTR promoter was labeled with fluorescein on the 5′ end of the forward strand to detect binding to the first κB site or the 5′ end of the reverse strand to detect binding to the second κB site. *B* and *C*, binding of p50/RelA_RHD_ to the HIV LTR DNA labeled on the 5′ end of forward (*B*) or reverse (*C*) strand was detected using fluorescence anisotropy and fit well to a model in which it binds each site independently with a *K*_*d*_ of 1.7 ± 0.4 nM. *D* and *E*, binding of p50/RelA_FL_ to the HIV LTR DNA labeled on the 5′ end of forward (*D*) or reverse (*E*) strand was detected using fluorescence anisotropy and fit well to a model in which it binds each site independently with a *K*_*d*_ of 1.3 ± 0.4 nM. *F*, dsDNA containing the sequence of the *NFKBIA* promoter was labeled with fluorescein on the 5′ end of the forward or reverse strand to detect binding to the first or second κB site, respectively. *G* and *H*, binding of p50/RelA_RHD_ to the *NFKBIA* DNA labeled on the 5′ end of forward (*G*) or reverse (*H*) strand was detected using fluorescence anisotropy and fit to a model in which it binds each κB site independently, with a *K*_*d*_ of 1.7 ± 0.5 nM for site 1 and 10 ± 2 nM for site 2. *I* and *J*, binding of p50/RelA_FL_ to the *NFKBIA* DNA labeled on the 5′ end of forward (*I*) or reverse (*J*) strand was detected using fluorescence anisotropy and fit to a model in which it binds each κB site independently, with a *K*_*d*_ of 1.5 ± 0.7 nM for site 1 and 5.1 ± 0.4 nM for site 2. Data points shown in this figure are the mean and SD of three independent experiments. *K*_*d*_ values are the mean and SEM of the best-fit values determined for each of the three experiments.

Using this approach, we monitored binding to both κB sites of the HIV LTR sequence and fit the anisotropy data to a simple model in which NFκB bound both sites independently (without positive or negative cooperativity) and with the same affinity (Fig. 7*A*). The data for both p50/RelA_FL_ and p50/RelA_RHD_ fit this model well, with *K*_*d*_ values of 1.3 ± 0.4 nM and 1.7 ± 0.4 nM, respectively (Fig. 7, *B*–*E*).

We next monitored binding of NFκB to the more complex *NFKBIA* promoter sequence containing tandem κB binding sites. In this case, to obtain a good fit, the fluorescence anisotropy data required a more complex model in which the two sites had different *K*_*d*_ values but exhibited neither positive nor negative cooperativity (Fig. 7*F*). For p50/RelA_FL_, the best-fit *K*_*d*_ for site 1 was 1.5 ± 0.7 nM and the best-fit *K*_*d*_ for site 2 was 5.1 ± 0.4 nM. For p50/RelA_RHD_, the best-fit *K*_*d*_ for site 1 was 1.7 ± 0.5 nM and the best-fit *K*_*d*_ for site 2 was 10 ± 2 nM (Fig. 7, *G*–*J*). The binding curves for site 2 were sigmoidal in nature, particularly for p50/RelA_RHD_. This reflects the lower binding affinity for this site and preferential binding to site 1 when the concentration of NFκB is below the concentration of κB binding sites (10 nM).

Overall, the results of these experiments recapitulate the patterns observed using DNA hairpins. For each κB site, the binding of p50/RelA_FL_ has slightly higher affinity on average than p50/RelA_RHD_. This was statistically significant for site 2 of the *NFKBIA* promoter, which has a noncanonical half-site sequence (*p* = 0.026) but not statistically significant for the full consensus sequences, site 1 of *NFKBIA* and HIV LTR (*p* > 0.4). The data for each tandem sequence fit well to a simple noncooperative model in which binding to either site is not influenced by binding to the other site. We did not find evidence for either positive or negative cooperativity using this approach, consistent with the EMSA results. The binding affinities were generally higher than those measured using hairpin DNA, which may be due to the presence of flanking DNA sequences and enhanced stability of longer dsDNA segments or to the location of the fluorophores relative to the κB sites. Importantly, the differences in binding affinities and the relative binding affinities are consistent in both the hairpin studies and the studies on tandem sequences.

## Discussion

The results presented here clearly demonstrate that the RelA TAD enhances binding of the p50/RelA heterodimer to nonspecific DNA sequences. This results in higher order complex formation with long DNA segments containing tandem κB sites and has important implications for our understanding of how the p50/RelA heterodimer engages nuclear DNA and regulates transcription. Multiple studies have found that one third to one half of the genomic sites bound by NFκB do not contain a κB motif (35, 36, 37, 38, 39, 40), highlighting the importance and physiological relevance of understanding how NFκB engages both consensus and nonconsensus DNA.

The only other protein for which the influence of the intrinsically disordered TAD on DNA-binding affinity and specificity has been measured is p53. Our results show that the RelA TAD has the opposite effect of the p53 TAD. First, the presence of the p53 TAD results in a decrease in binding affinity for nonconsensus DNA relative to the DNA-binding domain alone, whereas the RelA TAD improves binding affinity over that of p50/RelA_RHD_ in all cases. Remarkably, the presence of the RelA TAD dramatically improves binding to nonconsensus DNA. Whereas, the p53 DNA-binding domain binds nonspecific DNA with a *K*_*d*_ of 65 nM, the p50/RelA_RHD_ binds nonspecific DNA with a *K*_*d*_ of 1500 nM (Table 3). The p53 TAD reduces affinity for nonspecific DNA by 5.7-fold, whereas the RelA TAD improves binding to nonspecific DNA by almost 12-fold (12). These changes in affinity result in the binding affinity of p50/RelA_FL_ for nonspecific DNA being even stronger (130 nM) than that for full-length p53 (370 nM).

**Table 3:** *K*_*d*_ values of p53^a^ and p50/RelA for consensus and non-consensus DNA

Protein construct	Nonconsensus DNA *K*_*d*_	Consensus DNA *K*_*d*_
p50/RelA_FL_	130 ± 40 nM	1–5 nM
p50/RelA_RHD_	1500 ± 100 nM	2–11 nM
p53 DBD + TAD	370 ± 30 nM	16 ± 2 nM
p53 DBD	65 ± 7 nM	18 ± 3 nM

a Values for p53 were obtained from Krois *et. al* (12).

The compact but disordered nature of the RelA TAD is similar to other acidic TADs in human transcription factors in which interactions between hydrophobic residues facilitate compaction while negatively charged residues prevent folding and loss of binding motif accessibility (5, 6, 53). Negatively charged intrinsically disordered regions are over-represented among nucleic acid–related proteins, and it has been proposed that their similarity to negatively charged nucleic acids enables autoinhibition of nucleic acid binding under some conditions (58). Indeed, the p53 TAD is thought to decrease the binding affinity for nonspecific DNA by interacting with the positively charged DNA-binding domain in an autoinhibitory manner (12). Additional experiments are needed to determine why the RelA TAD has the opposite effect. Notably, although the TA1 and TA2 motifs of the RelA TAD are compositionally similar to other acidic TADs such as the p53 TAD, the long region between the RHD and TA2 (residues 319–424) is compositionally distinct, with a net charge of +2 and containing 24% proline residues. It is possible that this region could contribute to the novel function we have discovered for the RelA TAD in enhancing binding to nonspecific DNA.

The cellular concentrations of p50 and RelA have been measured in the ∼200 to 400 nM range in mouse embryonic fibroblasts and B-cells (59). Given the expected nuclear volume of 10% of the cell volume (60), the concentration of p50/RelA in the nucleus would be 2 to 4 μM after a strong activating signal. This greatly exceeds the measured binding affinity of 130 nM for p50/RelA_FL_ to nonconsensus DNA, and under these conditions, nearly all p50/RelA heterodimers would be bound to DNA. Given the presence of only 10^3^ to 10^4^ consensus κB sites in the human genome, many of the NFκB dimers would be bound to imperfect sites or nonspecific DNA sequences (39, 40, 41). A recent paper that tracked single molecule transcription factor dynamics in the nucleus found that several different transcription factors had a wide distribution of dwell times, best described by a continuum of affinities for DNA rather than simple specific *versus* nonspecific binding (61). The observation that p50/RelA_FL_ can bind nonspecific or imperfect DNA sequences with moderate affinity is consistent with a continuum of binding affinities and likely beneficial to transcription factor function. This is consistent with theoretical studies that show disordered regions of transcription factors can facilitate partially bound states and binding to nonspecific sequences (62). One possibility is that its binding site search would be more efficient if interactions with nonspecific DNA reduced the dimensionality of the search.

Transcription activation by the p50/RelA heterodimer occurs quickly following inflammatory signaling, as NFκB dimers flood the nucleus within the first 15 min. Although only weak ChIP-Seq peaks are generally detected within the first 10 to 15 min following inflammatory stimulation, widespread nucleosome repositioning has been observed during this time period prior to the appearance of robust ChIP-Seq peaks at 30 min (63, 64). Consistent with this *in vivo* result, we recently found that the p50/RelA_RHD_ is capable of binding nucleosomes and unraveling nucleosomal DNA *in vitro* through interactions with both specific κB sequences and nonspecific regions (65). We predict that p50/RelA_FL_ would be even more effective in opening nucleosomes, given its enhanced ability to bind nonspecific DNA sequences. This could have important implications for the search capabilities of p50/RelA, enabling it to more efficiently locate κB sites in both free and nucleosome-bound DNA in order to rapidly activate gene transcription.

The robust ChIP-Seq peaks observed at κB consensus sequences are clearly predicted based on the 1 to 5 nM binding affinity we measured for p50/RelA_FL_. The ChIP-Seq peaks at nonconsensus sequences may arise *via* a combination of the moderate DNA-binding affinity of ∼100 nM in addition to interactions with other proteins bound at these sites (35, 37). A recent study found that multiple human and yeast transcription factors undergo liquid–liquid phase separation mediated by their TADs *in vitro* when mixed with Mediator subunit MED1 (66). The phenomenon of biomolecular condensation *via* phase separation has been proposed as a general mechanism for efficient transcription regulation, particularly at nuclear super enhancer sites, although this is subject to ongoing debate (67, 68, 69). NFκB has not been shown to undergo liquid–liquid phase separation or biomolecular condensation in the nucleus but is known to associate strongly with super enhancer elements (63). In addition to its involvement in protein–protein interactions that could regulate the formation of large multiprotein assemblies such as super enhancers, the RelA TAD could also promote localization to these elements by facilitating interactions with nonspecific DNA within these nuclear sites. Although we did not find evidence for cooperativity between tandem κB motifs, the presence of multiple κB sites within gene regulatory regions could have an avidity effect in localizing NF-κB to these elements without requiring saturation of all κB sites.

This work expands on a growing body of literature regarding how intrinsically disordered domains can influence the activity of folded regions of proteins. Most prior studies of the effects of TADs on transcription factor–DNA binding have found that TADs lower DNA-binding affinity and/or increase specificity (7, 8, 9, 10, 11, 12, 13, 14). Our results provide an important counterexample in which the RelA TAD increases DNA-binding affinity, particularly for nonconsensus DNA sequences. Additional work is needed to elucidate the sequence and structural elements that determine the effects of TADs on transcription factor–DNA interactions and to understand how these effects relate to cellular protein function.

## Experimental procedures

### Protein expression and purification

N-terminal hexahistidine murine p50_39–350_/RelA_19–321_ (hereafter referred to as p50/RelA_RHD_) was expressed using a modified pET22b vector containing the genes for both polypeptides as described previously (70). The DNA for murine RelA residues 19 to 549 was synthesized and subcloned into a modified pET22b vector, which already contained the gene for N-terminal hexahistidine-p50_39-350_ (hereafter referred to as p50/RelA_FL_). The DNA sequence of the RelA_TAD_ (RelA residues 340–549) was subcloned into pET28a vector with a C-terminal hexahistidine tag.

All vectors were transformed into *E. coli* BL-21 (DE3) cells and grown to an *A*_600_ of 0.5 to 0.7 at 37 °C in M9 minimal media with antibiotic selection. Cultures were cooled on ice for 20 min and then protein expression was initiated by the addition of 0.2 mM IPTG. Cultures were incubated at 18 °C for 16 h and then harvested by centrifugation. Pellets were stored at −80 °C.

The p50/RelA_RHD_, p50/RelA_FL_, and RelA_TAD_ constructs were lysed by sonication and purified by Ni^2+^-NTA chromatography as described previously for p50/RelA_RHD_ (65). Following overnight dialysis, protein was aliquoted and stored at −80 °C. Prior to experiments, aliquots were thawed and further purified. p50/RelA_RHD_ and p50/RelA_FL_ were purified by cation exchange chromatography (MonoS; GE healthcare) to remove bound nucleic acids, as described previously (65). Protein was further purified by SEC using a Superdex 200 column (GE healthcare) in SEC buffer (25 mM Tris, 150 mM NaCl, 0.5 mM EDTA, 1 mM DTT, adjusted to pH 7.5 at room temperature [RT]). Care was taken to separate p50/RelA_FL_ from a breakdown product that eluted at the same volume as p50/RelA_RHD_. RelA_TAD_ was purified by SEC using a Superdex 75 column, followed by a Superdex 200 column (GE healthcare) in the same buffer.

All purification chromatography steps were conducted in a 4 °C cold room. Purity of all proteins was assessed by SDS-PAGE. The protein concentration was determined by absorption at 280 nm using a NanoDrop spectrophotometer. Purified protein was stored at 4 °C, and all experiments were conducted within 72 h of purification by SEC.

### Analytical SEC

Protein samples were prepared at two concentrations in SEC buffer. Both 5 and 10 μM samples of p50/RelA_FL_ and p50/RelA_RHD_ were used, and 10 and 20 μM samples of RelA_TAD_ were used due to its lower molar absorptivity at 280 nm. Samples were injected onto a Superdex 200 10/300 column (GE Life Sciences) equilibrated in the same buffer using a 100 μl sample loop at 4 °C.

### SAXS

SAXS data were collected at SIBYLS beamline 12.3.1 at the advanced light source following standard procedures (71). Three different concentrations (1.25 mg/ml, 2.5 mg/ml, and 5 mg/ml) of samples (RelA_TAD_, p50/RelA_RHD_, and p50/RelA_FL_) were prepared in SEC buffer. All SAXS data were analyzed using ATSAS. The scattering intensity of the buffer was subtracted from the sample, and the resultant intensity was used for analysis. The radius of gyration (R_g_) was calculated using the Guinier approximation. The pairwise distance distribution function P(r) was computed using the program GNOM with standard procedures. Data from all three concentrations were comparable, but only the lowest concentration (1.25 mg/ml) data were used in the analysis of all three proteins for direct comparisons.

The R_g_ of an excluded volume polymer containing 218 amino acid residues was calculated to be approximately 48 Å using the equation R_g_ = R_0_∗N^ν^, where R_0_ and ν have the values 1.927 Å and 0.598, respectively, as previously determined for denatured proteins in solution (50). The expected R_g_ of a globular protein of this size (18–20 Å) was estimated using the average R_g_ of proteins containing between 201 to 250 amino acid residues, based on structural characterization of 3412 globular proteins (51).

### Structural modeling based on the SAXS data

To generate a structural model of the RelA_TAD_ (residues 322–549), twenty independent *de novo* AWSEM structure prediction runs were performed to look for folded regions (52). During predictions, the forces guiding folding include backbone terms such as Ramachandran angle preferences, direct and water-mediated residue–residue contacts, residue burial preferences, hydrogen bonding in α-helices and β-sheets, long-range electrostatic interactions, and a bioinformatic local-in-sequence interaction known as the associative memory term (72). In each run, the temperature was cooled from 600 K to 100 K over 8 million 2 femtosecond time steps. To generate models of the exact RelA_TAD_ protein construct used for the SAXS data collection, residues 321 to 339 were removed and a C-terminal 6×His-tag was added using the web-based server AllosMod (73) which is integrated with the FoxS server for rigid body modeling of SAXS data. Next, BilboMD was used to find the conformations of RelA_TAD_ that best matched the SAXS data (74). In BilboMD, the helical regions predicted by AWSEM were held stable and the rest of the sequence was allowed to move. We generated 800 conformations at each R_g_ value using the R_g_ determined from the Guinier analysis ± 4 Å. The FoXs server was used to calculate the intensity profiles from the RelA_TAD_ conformations generated by BilboMD (75) and each was compared to the SAXS data to determine the best match. The best-fitting model had a Χ^2^ value of 1.1.

For p50/RelA_RHD_, we found that the open conformation we previously sampled with atomic MD (26) fit the SAXS data very well. BilboMD was used to further refine the structure using a similar approach as described for RelA_TAD_ and resulted in a model with a Χ^2^ value of 1.35.

To generate a model of p50/RelA_FL_, we docked (both PatchDock and FoXSDock gave similar results) the RelA_TAD_ model generated by AWSEM simulations onto the model of p50/RelA_RHD_ that best fit the SAXS data. Docking without IκBα present resulted in the TAD binding to the same face of the RHD as IκBα and resulted in models that did not fit the SAXS data well. Therefore, we aligned the dimerization domains of the p50/RelA_RHD_ SAXS model to a crystal structure of the dimerization domains bound by IκBα (Protein Data Bank 1NFI) (76) and included IκBα as a part of the complex during the docking in order to guide the placement of the TAD away from this site. Several hundred docked models were obtained. The IκBα molecule was removed, and Modeller was used to connect the TAD to the RelA RHD. We used FoXS to calculate the intensity profiles to find the 10 models that best agreed with the SAXS data and BilboMD to refine the models. Two models agreed best with Χ^2^ of less than 1.4.

### HDX-MS

Samples containing 5 μM p50/RelA_RHD_ or p50/RelA_FL_ alone or in the presence of 5 μM HIV-LTR hairpin DNA were prepared in SEC buffer. HDX-MS experiments were performed using a Waters Synapt G2Si time-of-flight mass spectrometer equipped with a nanoACUITY UPLC system with HDX technology and a LEAP autosampler. For each time point, 4 μl of sample was incubated at 25 °C for 5 min, then mixed with 56 μl D_2_O buffer (25 mM Tris, 150 mM NaCl, 1 mM DTT, and 0.5 mM EDTA, pH 7.5 at RT). Time points were collected in triplicate for 0, 10, 30, 60, and 120 s incubations in D_2_O buffer. Following incubation, 50 μl of the protein solution was mixed with 60 μl of quench solution (5 M GdnHCl, 0.5% formic acid) in a 1 °C sample chamber. The pH of this mixture was measured to be 2.7 on ice. The quenched protein solution was then injected onto an in-line pepsin column (immobilized pepsin, Pierce, Inc). The resulting peptides were trapped and then separated on a C18 column (Acquity UPLC BEH C18, 1.7 μM, 1.0 × 50 mm; Waters Corporation) using a 7% to 85% acetonitrile gradient with 0.1% formic acid over 7.5 min and directly electrosprayed into the mass spectrometer. The mass spectrometer was set to collect data in the Mobility ESI+ mode, with mass acquisition range of 200 to 2000 (m/z) and scan time of 0.4 s. Continuous lock mass correction was accomplished with an infusion of leu-enkephalin (m/z = 556.277) every 30 s (mass accuracy of 1 ppm for calibration standard). For peptide identification, the mass spectrometer was set to collect data in MS^E^, mobility ESI+ mode instead. The peptides were identified from triplicate MS^E^ analyses of 10 μM of protein solution, and data were analyzed using PLGS 2.5 (Waters Corporation). The peptides identified in PLGS were then analyzed in DynamX 3.0 (Waters Corporation). The relative deuterium uptake for each peptide was calculated by comparing the centroids of the mass envelopes of the deuterated samples *versus* the undeuterated controls following previously published methods (77). The deuterium uptake was corrected for back exchange as previously described (78). Deuterium uptake plots were generated in DECA (github.com/komiveslab/DECA), and the data are fitted with an exponential curve for ease of viewing (78). Community guidelines have been followed, and the data are publicly available on the Massive data repository (79).

### Fluorescence anisotropy DNA-binding assay

The DNA oligonucleotides listed below were purchased from Integrated DNA Technologies with a 5′ 5AmMC6 modification. After resuspension in water, approximately 20 nmol DNA was mixed with 300 nmol fluorescein isothiocyanate (Sigma) in a final volume of 100 μl in Borax buffer (0.1 M sodium tetraborate pH 8.5). Samples were incubated at 70 °C for 6 h, then 500 μl 100% ethanol was added, and samples were stored at −20 °C overnight to precipitate the DNA. Precipitated DNA was pelleted by centrifugation, then purified *via* reverse-phase HPLC as described previously (56). Solvent was removed using a SpeedVac. Hairpin DNA sequences used for the experiments were as follows. dsDNA sequences are reported in Figure 7.

IFN-β: 5′ GGGAAATTCCTCCCCCAGGAATTTCCC 3′

Urokinase (UK): 5′ GGGAAAGTACTCCCCCAGTACTTTCCC 3′

RANTES: 5′ GGGAGTTTCCTCCCCCAGGAAACTCCC 3′

HIV-LTR: 5′ GGGACTTTCCTCCCCCAGGAAAGTCCC 3′

Random: 5′ GTAGACGTGCTCCCCCAGCACGTCTAC 3′

NFKBIA site 1: 5′ TGGAAATTCCCTCCCCCAGGGAATTTCCA 3′

NFKBIA site 2: 5′ AGAGAAATCCCTCCCCCAGGGATTTCTCT 3′

Labeled DNA samples were resuspended in TE buffer, and DNA concentration and labeling efficiency were determined by measuring the absorbance at 260 nm and 495 nm on a NanoDrop spectrophotometer. dsDNA was created by mixing the labeled strand with an equimolar concentration of the unlabeled complementary strand and incubating in a 95 °C heat block for 5 min before turning off the heat block and allowing the strands to anneal as the sample to slowly cooled to RT over the course of 2 to 3 h.

Fluorescein-labeled DNA (5 nM) was mixed with varying concentrations of p50/RelA_RHD_ and p50/RelA_FL_ in triplicate in a black 96-well plate and incubated at RT for 2.5 to 3 h. The fluorescence anisotropy was measured at 25 °C on a Beckman Coulter DTX 880 Multimode plate reader. The excitation and emission wavelengths were 495 nm and 519 nm, respectively. The integration time of the data collection was 1 s. Anisotropy was calculated using the equation r = [I(V,V) − GI(V,H)]/[I(V,V) − 2GI(V,H)], where r is anisotropy, I(V,V) is the fluorescence intensity in the parallel direction, I(V,H) is the fluorescence intensity in the perpendicular direction, and G is the grating factor. The G-factor used for calculating the anisotropy is 0.67, as previously determined for this instrument.

Fluorescence anisotropy data for the hairpin κB DNA sequences were fit to the following equation to determine the equilibrium binding affinity: $\text{y}=\text{A}\left({\left[\text{DNA}\right]}_{0}+\text{x}+{\text{K}}_{\text{d}}-\sqrt{{\left({\left[\text{DNA}\right]}_{0}+\text{x}+{\text{K}}_{\text{d}}\right)}^{2}-4{\left[\text{DNA}\right]}_{0}\text{x}}\right)/2{\left[\text{DNA}\right]}_{0}$, where y is the change in fluorescence anisotropy, A is the maximum change in fluorescence anisotropy, x is the varying concentration of p50/RelA, [DNA]_0_ is the concentration of DNA (5 nM), and K_d_ is the equilibrium binding affinity (80). Anisotropy data for the random hairpin DNA sequence were fit to the equation $\text{y}=\text{A}*\text{x}/\left(\text{x}+{\text{K}}_{\text{d}}\right)$.

DNA-binding assays were conducted on three separate days with different DNA and protein preparations. For each assay, data were fit to the aforementioned equations to determine the *K*_*d*_. The *K*_*d*_ values reported in Figure 6 are the mean and SEM of the *K*_*d*_ values determined for each of the three replicate experiments. Due to slight variability in the plateau anisotropy value between different DNA preparations, data from each experiment were normalized by the value of A determined for that experiment in order to generate the plots in Figure 6, *A*–*G* showing the averaged data from the three experiments.

Due to the presence of two binding sites, the fluorescence anisotropy data obtained using DNA sequences with tandem κB sites could not be fit to the equations reported before for single site binding. Instead, they were fit using an ODE MATLAB-based approach similar to that described previously (81). Detailed equations and approach are described in the supporting information.

### EMSAs

Samples were prepared in SEC buffer and diluted 1:1 in 2× EMSA loading buffer (40 mM Tris pH 7.5 at RT, 100 mM NaCl, 2 mM MgCl_2_, 2 mM DTT, 0.5 mg/ml bovine serum albumin, 10% glycerol (v/v), and 0.01% bromophenol blue (w/v)). The final DNA concentration was 250 nM, and the final protein concentrations were 0, 62.5, 125, 250, 500, 1000, and 2000 nM for gels in Figures 3 and 4. For the competition experiment in Figure 5, the final dsDNA and hairpin DNA concentrations were 250 nM and the final protein concentration was 500 nM. Samples were incubated for 1 h at RT, then run on 5% polyacrylamide TBE gels in 0.5× TBE buffer in a 4 °C cold room. Gels were stained using SYBR Gold nucleic acid stain and imaged using a Typhoon imager. Band intensities were quantified using ImageJ (https://imagej.nih.gov/ij/), and mean and SD were calculated for two biological replicates using different protein preparations.

## Data availability

SAXS data is available at SASBDB.org; p50/RelA_RHD_ (SASDHB5), p50/RelA_FL_ (SASDHC5), and RelA_TAD_ alone (SASDHD5). These data are available at the following URLs:

https://www.sasbdb.org/data/SASDHB5/3wubsouaf0/ https://www.sasbdb.org/data/SASDHC5/xwlllmre95/ https://www.sasbdb.org/data/SASDHD5/4x8i7qpqzi/

The full project summary is available at https://www.sasbdb.org/project/967/5isz7mjm7q/

The raw HDXMS data files and analyzed data are available at massive.ucsd.edu dataset MSV000089247. Uptake plots may be generated from the state data excel file using the DECA program available at https://github.com/komiveslab/DECA (78).

## Supporting information

This article contains supporting information.

## Conflict of interest

The authors declare that they have no conflicts of interest with the contents of this article.
